# Construction of a pre-service training program for nursing assistants in China

**DOI:** 10.1186/s12909-026-09925-x

**Published:** 2026-07-20

**Authors:** Hong Feng, Wei Ding, Xiaoyan Chen, Caoli Wen, Mengqi Li, Fen Hu, Bilong Feng

**Affiliations:** 1 Nursing Department, Zhongnan Hospital of Wuhan University, 169 Donghu Road, Wuchang District, Wuhan, 430071 China; 2 Centre for Health Research Methodology, School of Nursing, Midwifery and Evidence Science, University of Galway, Galway, Ireland; 3 School of Nursing and Health, Zhengzhou University, Zhengzhou, China

**Keywords:** Nursing assistants, Training program, Analytic Hierarchy Process

## Abstract

**Background:**

Nursing assistants (NAs) are a key occupational group that provide daily living care and nursing support across medical and integrated healthcare settings. NAs have been introduced to alleviate nursing shortages and better meet patients’ care needs. In many countries, mandatory pre-employment training programs are tailored to the NAs’ role. However, China lacks a standardized pre-service training program, leading to fragmented content and variable training quality. In response to national policy priorities, this study aims to develop a standardized pre-service training program for NAs in China.

**Methods:**

A modified Delphi method and the Analytic Hierarchy Process (AHP) were used to establish a competency-based pre-service training program, guided by the Competency Iceberg Model, from June to October 2022. An expert panel (*n* = 12) from nine tertiary hospitals across eight provinces participated in two rounds of Delphi consultations to reach consensus on program content. Expert agreement was evaluated using consensus levels and Kendall’s W coefficient.

**Results:**

The final training program comprises a three-level framework with three first-level indicators, 25 s-level indicators, and 100 third-level indicators, encompassing both practical care competencies and latent professional competencies. Expert consensus was achieved across all hierarchical indicators. Kendall’s W values for the second- and third-level indicators were 0.222 and 0.179, respectively, both of which were statistically significant (*p* < 0.001). The AHP results assigned the highest priority to skills (0.50), followed by professionalism (0.25) and knowledge (0.25). Within these domains, practical care skills, including mobility care, sputum management, and latent competencies, including emotional management and communication, received the highest priority weightings. These weightings can provide an evidence-informed rationale for prioritising training content, allocating instructional time, and further guiding practical implementation.

**Conclusion:**

This study established a structured, competency-based pre-service training program for NAs by integrating expert consensus with quantitative prioritization. The program provides a holistic framework and evidence-informed basis for developing NAs training and standardizing pre-service training in Chinese healthcare settings. Future research should examine its feasibility, effectiveness, and implementation across diverse clinical contexts.

## Background

To alleviate global nurse shortages and better meet rising healthcare demands [1], assistive nursing roles have become increasingly prominent in healthcare systems [2, 3]. Their designations vary across systems and include unlicensed assistive personnel in the United States, healthcare assistants in the United Kingdom, and nursing assistants (NAs) in Australia. These workers are typically defined as unlicensed or minimally regulated personnel who provide basic patient care, including assistance with activities of daily living, vital signs monitoring, and supportive care, under the supervision of registered nurses or midwives [4]. In China, *the National Occupational Standards for Nursing Assistants (2024 Edition)* defined NAs as personnel who provide daily living assistance and auxiliary nursing services, while explicitly excluding technical nursing procedures from their scope of practice [5]. This regulatory boundary directly shapes training needs. Unlike registered nurses, who provide specialized care and participate in clinical decision-making, NAs focus on foundational supportive care. This division of labor requires a training program that is distinct from nursing education and aligned with the defined NAs’ scope of practice.

Although stakeholders recognize the value of NAs [6, 7], these benefits depend largely on whether NAs possess adequate professional competencies [8–10]. When such competencies are insufficient, basic care responsibilities may shift to registered nurses, increasing workload and supervisory demands, reducing time available for specialized clinical care, and delaying timely interventions. These effects may compromise care quality and pose risks to patient safety [11].

Broad international consensus exists on the need for pre-service training to ensure baseline competency [3, 6, 12]. However, a recent scoping review [13] highlighted significant fragmentation in global practice. There is no consistent agreement on terminology, role boundaries, or scope of practice for NAs, and standardized pre-service training frameworks remain undeveloped. Training pathways vary across countries, reflecting differences in healthcare system and role definitions [13]. For example, in the United States, federal regulations require classroom and clinical training covering basic care activities, communication, personal care, professionalism, and safety protocols [14], with some states including additional topics such as workplace problem-solving, work planning and organization, and occupational protection [14, 15]. In the United Kingdom, training frameworks emphasize the delivery and monitoring of planned care, recognition of individual needs, basic life support, and infection prevention and control, with an emphasis on evidence-based practice [16]. Similarly, several European countries focus on specialized knowledge related to specific care areas and patient groups [3]. Despite these developments, a global unified competency-based training framework for NAs has yet to be established.

In China, the development of the NAs role has followed a distinct, policy-driven pathway. NAs were officially recognized as an occupational category in 2015 and established as an independent profession in 2022. NAs primarily work in healthcare institutions and integrated medical-care facilities. However, many are recruited by patients’ families or through intermediary agencies and receive little or no formal training [17]. This can limit their ability to meet diverse care needs and increases potential safety risks [18, 19]. Consequently, the need for structured NAs training is widely recognized [20, 21]. Although previous studies have attempted to develop training programs [22, 23], substantial variation in content persists, reflecting a lack of consensus on core competencies. For example, some training programs include clinical monitoring skills [24], whereas others exclude them [22], underscoring the absence of a unified, evidence-based training framework.

In response, the National Health Commission of China issued the *Notice on Strengthening Training and Standardized Management of Nursing Assistants*, which mandates minimum training hours and essential content [25]. This policy provides a foundation for standardisation. Subsequently, provinces have developed local training programs based on their interpretations of the national guidance. Regional pilots highlighted diverse emphases in training program structure and instructional methods [26, 27]. While these initiatives offer practical insights, they also reveal limitations in the national framework. Without specified content prioritization or weighting, regional programs differ in scope, depth of coverage, and allocation of training time, potentially hindering consistency in training quality.

Therefore, this study aims to develop a structured, competency-based pre-service training program for NAs in China. The research is guided by McClelland’s Competency Iceberg Model, which distinguishes between explicit and implicit competencies and emphasizes the foundational role of implicit competencies in sustained job performance [28]. Based on this model, we constructed a three-dimensional training framework encompassing skills, knowledge, and professionalism. To establish and prioritize the competency indicators, we employed a modified Delphi method in combination with the Analytic Hierarchy Process (AHP). The modified Delphi approach was used to achieve structured expert consensus through iterative anonymous consultations, while AHP was subsequently applied to quantify the relative weight of each indicator, thereby informing training program prioritization and allocation of instructional resources.

## Methods

### Design

Grounded in McClelland’s Competency Iceberg Model [28], this study employed a modified Delphi method combined with the AHP to construct a pre-service training program for NAs. The Competency Iceberg Model categorizes individual competencies into overt competencies above the surface, such as knowledge and skills, and latent competencies below the surface, such as social roles, self-concept, traits, and motives. Overt competencies represent the explicit requirements for task performance, whereas latent competencies are considered pivotal in driving superior job performance and long-term professional development. Accordingly, the training program in this study was structured into three core modules: knowledge, skills, and professionalism.

- The knowledge and skills modules correspond to overt competencies and represent the explicit, observable, and directly trainable requirements for performing NAs tasks.
- The professionalism module addresses the latent competencies, encompassing social roles, self-concept, traits, and motives that are fundamental to sustained high-quality performance and professional conduct.

To operationalize these latent competencies into teachable and assessable training program components, the professionalism module was further delineated into two secondary dimensions. First, humanistic literacy focuses on intrinsic self-concept and traits, including psychological characteristics and emotional management. These elements underpin a NA’s capacity for empathy, resilience, and self-regulation in demanding care environments. Second, professional quality captures the internalization of expected social roles and motives within the healthcare context. It is reflected in indicators such as responsibility, ethics, legal awareness, and behavioral codes, thereby ensuring alignment with professional norms and societal expectations.

Consequently, this framework is designed to comprehensively target that the training program comprehensively targets the “what to do” (knowledge), “how to do” (skills), and “how to be” (professionalism). This approach yields a holistic competency-based training program grounded in established theoretical constructs.

**Fig. 1:**
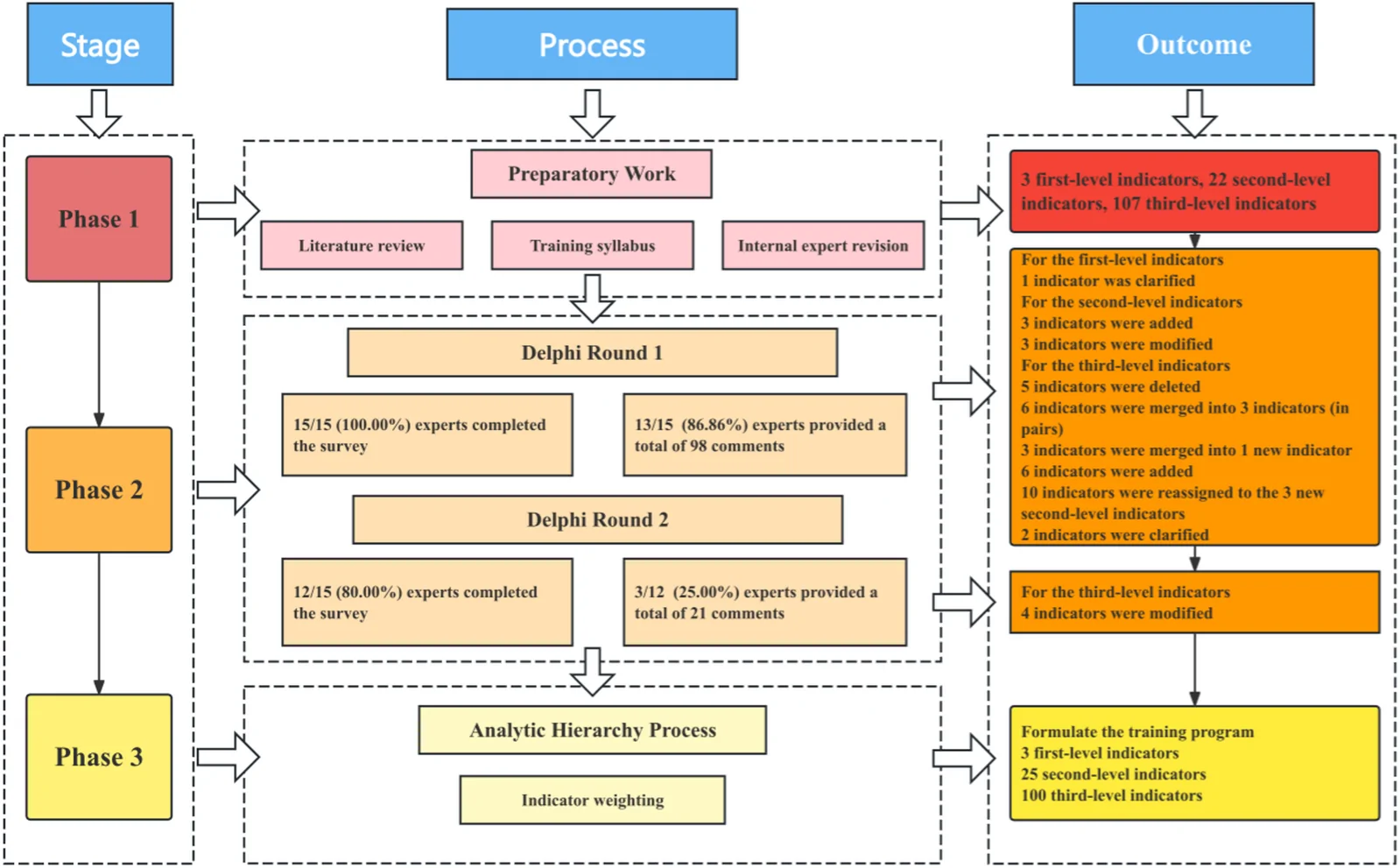
Flow chart of the research process

This study was conducted from June to October 2022. The Delphi method was employed to reach consensus on the training program content, and AHP was applied to assign hierarchical weights to the evaluation indicators through pairwise comparisons and consistency checks. A flow chart illustrating the entire research process is shown in Fig. 1.

### Establishment of the research team

The research team consisted of six members: two chief nursing officers (a Director of Nursing Education and a Nursing Department Manager), one nursing education specialist, one nursing management specialist, and two medical postgraduate students. Both chief nursing officers and the nursing management specialist each had more than 10 years of experience in registered nurse management and training program development, providing domain-specific expertise for study design and indicator refinement. All team members received prior training in the modified Delphi method and AHP before the study initiation. The primary tasks of the team were to: (1) conduct literature reviews and develop the draft training program; (2) design the consultation questionnaire; (3) recruit consulting experts, manage the consultation process, and distribute the questionnaire; and (4) summarize and analyze expert feedback, and iteratively revise the program content.

### Generation of the initial draft training program

Guided by the Competency Iceberg Model, two postgraduate students, under the supervision of the nursing education specialist, conducted a comprehensive review to generate an initial indicator pool. This included: (1) Policy analysis of key documents (e.g., *Notice on Strengthening Training and Standardized Management of Medical Care Attendants*, *the National Occupational Standards for Medical Care Workers (2024 Edition*)); (2) Review of training syllabi from three regional pilot programs; (3) Systematic literature search of CNKI and PubMed using terms “nursing assistant” and “training”; and (4) Analysis of two widely used NAs training textbooks. Potential competency statements were extracted from these sources and inductively categorized to generate the initial draft competency framework. This process resulted in three first-level, 23 s-level, and 106 third-level candidate indicators. Subsequently, three internal experts (two chief superintendent nurses and one NAs instructor) evaluated these indicators, providing 12 recommendations: eight related to wording clarification, three to item merging, and one to the addition of a new indicator. After the research team consolidated overlapping items, the refined pool consisted of three potential first-level indicators, 22 potential second-level indicators, and 107 potential third-level indicators.

### Development of the expert consultation questionnaire

A structured expert consultation questionnaire was designed to guide the consultation process. The questionnaire consisted of three core sections: (1) An introduction of the overall survey, the draft training program, and a request for personal information; (2) Experts were required to evaluate the importance of each item on a five-point Likert scale from 1 (not important) to 5 (very important). An open-ended column was provided after each item for experts for qualitative feedback; and (3) The expert self-assessment form consisted of two sections, a demographic questionnaire (collecting information such as age, educational background, years of experience, and professional title) and a self-rated scale measuring their familiarity with the research content. The second-round questionnaire integrated findings from the first round, allowing experts to review the aggregate results and further refine consensus.

### Selection of the expert panel

A purposive sample of 15 experts was invited, consistent with the recommended panel size for Delphi studies [29]. Experts were eligible if they: (1) had more than 10 years of experience as nursing professionals; (2) specialized in nursing management, nursing education, or frontline clinical practice; (3) held a bachelor’s degree or higher; and (4) had at least two years of experience in NAs management or training. Experts were recruited from Beijing, Hubei, Henan, Sichuan, Shanghai, and Guangzhou provinces. Senior nursing professionals from tertiary hospitals were purposively selected because they hold primary responsibility for supervising and training NAs in China and have comprehensive insight into the competencies required for safe and effective practice within the existing regulatory framework.

### Delphi process

The two-round Delphi survey was conducted between June and August 2022. Potential experts were approached by the corresponding author via email or WeChat. The corresponding author explained the study purpose and invited participation. Upon obtaining informed consent, consultation questionnaires were distributed via email or WeChat. Each Delphi round lasted two weeks, with reminders sent by email or WeChat three days before the deadline to experts who had not yet completed the questionnaire. The average completion time for each round was approximately 12 days. After each round, the research team screened the items using the following criteria: (1) coefficient of variation (CV) ≤ 0.25; (2) mean importance score ≥ 3.50; and (3) expert consensus level ≥ 75%. Items failing to meet these criteria were modified, removed, or added based on the research team’s analysis of expert feedback.

### Analytic hierarchy process

AHP was used to calculate indicator weights. The mean differences in second-round importance scores were converted into Saaty’s fundamental nine-point scale [30]. A three-level hierarchical model was constructed, including the first level, represented by the training program indicator system; the second level, represented by the training program dimensions such as theoretical knowledge and operational skills; and the third level, represented by specific training program items. Subsequently, hierarchical single sorting and consistency testing were performed using Microsoft Excel to calculate initial weights. These weights were normalized to obtain the weight vector, followed by calculation of the maximum eigenvalue (λ_max) and consistency index (CI), where CI = (λ_max - *n*) / (*n* − 1) and *n* denotes the order of the judgment matrix. The consistency ratio (CR) was then derived as CR = CI / RI, where RI denotes the random consistency index corresponding to the matrix order. A CR value less than 0.10 indicated acceptable consistency [31]. Matrices failing to meet this threshold were revised and recalculated to ensure reliability. Finally, hierarchical total sorting and overall consistency test were performed. Weighted summation was implemented using Microsoft Excel to obtain the comprehensive weight of each item at the third level relative to the first level. The overall CR was calculated simultaneously, with an overall CR < 0.10 indicating that the overall judgment matrix passed the consistency test.

### Data analysis

Statistical analyses were performed using Microsoft Excel and IBM SPSS version 27.0. Descriptive statistics were used to analyze frequency, proportion, mean and standard deviation. The recovery rate (RR) was calculated to test expert engagement, with RR > 70% indicating acceptable effectiveness [32]. The degree of expert authority was expressed using the authority coefficient (Cr), calculated as the arithmetic mean of the judgment coefficient (Ca) and familiarity coefficient (Cs), where Cr = (Cs + Ca) / 2. A Cr ≥ 0.70 was considered reliable and indicated a high level of expert authority [33]. The consistency of expert opinions was assessed using the coefficient of variation (CV) and Kendall’s coefficient of concordance (W). The CV reflects variation in experts’ importance ratings for specific training program indicators, with CV < 0.25 generally considered acceptable. Kendall’s W and its associated significance test were used to determine the degree of consistency across consulting experts in rating of the training program indicators, with values close to 1 indicating stronger agreement [34]. A value of *P* < 0.05 indicated statistical significance.

### Study rigor

To ensure methodological rigor, the research team provided all invited experts with detailed study objectives and explanatory materials prior to the consultation. Upon questionnaire collection, the team first reviewed all responses for completeness. Questionnaires with more than 10% missing data were excluded. Dual independent data entry with cross-validation was employed to ensure data integrity. None of the returned questionnaires had more than 10% missing data, therefore, no questionnaire was excluded on this basis (0/27, 0%). Qualitative feedback provided in the survey was synthesized through collective discussion by the research team. Final decisions were based on a synthesis of expert judgement and evidence from the relevant literature.

### Ethical approval

This study involved expert consultation only, with no patient participation or clinical intervention, and was therefore exempt from formal ethical review. The exemption was confirmed by the local Ethics Committee, and no approval number was issued. All procedures were conducted strictly in accordance with the principles of the Declaration of Helsinki (2024 revision).

All participants provided written informed consent prior to participation. The consent form was printed, signed by each expert, and returned to the research team electronically as a scanned copy. Participation was voluntary. To ensure anonymity and confidentiality, the following procedures were implemented: (1) Each completed electronic questionnaire was assigned a unique study ID upon receipt. Each expert’s name and contact information were stored in a separate, password‑protected file accessible only to the principal investigator (FBL). (2) All electronic data were stored on an encrypted, password‑protected institutional computer. Access to raw data was restricted to three research team members (HF, FH, DW). (3) No individual expert responses were disclosed to other experts or to anyone outside the research team.

## Results

### Demographic characteristics of the expert panel

The expert panel comprised 15 members in the first round and 12 members in the second round. The 12 experts who completed the second round represented nine tertiary hospitals across seven provinces and municipalities, including Sichuan, Beijing, Shanghai, Henan, Hubei, Hunan, and Guangdong. All experts had more than 10 years of experience in direct clinical supervision, training program development, or NAs management. The panel was predominantly female and included experts with advanced professional titles and educational backgrounds. Detailed demographic characteristics of the expert panel are presented in Table 1.

**Table 1:** Demographics of the expert panel

Characteristics	First round *n* (%) (*n* = 15)	Second round *n* (%) *(n* = 12)
Age (years)		
41–45	4 (26.67)	3 (25.00)
46–50	4 (26.67)	4 (33.33)
> 51	7 (46.67)	5 (41.67)
Educational background		
Bachelor’s degree	9 (60.00)	7 (58.33)
Master’s degree	4 (26.67)	3 (25.00)
Doctoral degree	2 (13.34)	2 (16.67)
Professional titles		
Intermediate	1 (6.67)	1 (8.33)
Associate senior	6 (40.00)	5 (41.67)
Full senior	8 (53.33)	6 (50.00)
Specialization		
Nurse education managers	7 (46.67)	6 (50.00)
Clinical nurse educators	2 (13.34)	1 (8.34)
Nurse managers	6 (40.00)	5 (41.67)
Postgraduate supervisor status		
Yes	7 (46.67)	5 (41.67)
No	8 (53.33)	7 (58.33)

### Response rates and expert engagement

Two rounds of Delphi consultation were conducted, with 15 questionnaires distributed in each round. In the first round, all 15 experts completed the questionnaire, yielding a response rate of 100%. A total of 98 comments were received from 13 (87%) experts. In the second round, 12 of the 15 experts completed the questionnaire, yielding a response rate of 80%. A total of 21 comments were received from six (50%) experts.

### Reliability of the expert panel

The authority coefficients of the expert panel were 0.95 in the first round and 0.94 in the second round, indicating a high level of expert authority. Consensus improved across rounds: Kendall’s W for third-level indicators increased from 0.119 in the first round to 0.179 in the second round. All items had a CV of less than 0.25, and Kendall’s W values were statistically significant (*p* < 0.05). These results indicate that the consultation had.

acceptable coordination and reliability, as shown in Tables 2 and 3.

**Table 2:** The result of expert opinions’ concentration degree

Delphi round	Hierarchical level	Number of indicators	Kendall’s W	x^2^	*P*-value
Round 1	First level	3	-	-	
	Second level	22	0.112	35.26	0.026
	Third level	107	0.119	188.44	< 0.001
Round 2	First level	3	-	-	-
	Second level	25	0.222	64.03	< 0.001
	Third level	100	0.179	201.86	< 0.001

### Revision and refinement of the indicator system

During the first round, the research team refined the indicator system based on expert feedback and predefined statistical criteria. At the first level, the indicator “attainment” was revised to “professionalism”. In the Chinese linguistic context, “professionalism” more accurately captures the latent competencies of the Iceberg Model, including social roles, self‑concept, traits, and motives. At the second level, three new indicators were added to distinguish more clearly between the skills and knowledge dimensions. The wording of three existing items was also revised to clarify their semantic meaning. At the third level, ten items were reassigned to the new second-level indicators and six similar items were merged into three consolidated indicators. The wording of two additional items was revised for greater precision. Meanwhile, three items with a CV > 0.25 and a consensus level < 75% were deleted, along with two additional items that failed to meet the 75% consensus threshold. In addition, six indicators, including the Heimlich Maneuver and Fire Emergency Drill, were added because of their practical importance. Similarly, the indicators “emergency response to water outage” and “emergency response to power outage” were retained despite exceeding the predefined CV threshold, as the research team deemed them to have substantial practical significance. In the second round, four items were further refined. For example, “the relevant indicators of vital signs” was changed to “knowledge of vital signs” to improve theoretical clarity. Therefore, the final training program consisted of three domains, 25 s-level indicators, and 100 third-level indicators.

### AHP weighting results of the competency framework

A total of 22 judgment matrices were formed, all of which had CRs < 0.1. The CRs for the first-level matrix was < 0.001. The CRs for the second-level matrices were < 0.001, 0.0412, 0.0083, respectively. The CRs for the third-level matrices ranged from < 0.001 to 0.0871. These results indicate that all judgement matrices met the consistency requirement. The detailed combined weights were presented in Table 3.

**Table 3:** Final training program for NAs and corresponding weights

First level		Second level		Third level		Mean (SD)	Coefficient of variation
Indicators	Weight	Indicators	Weight	Indicators	Combined weight		
Professionalism	0.25	Humanistic literacy	0.333	Psychological traits	0.025	4.92 ± 0.29	0.059
				Emotional management	0.058	4.83 ± 0.39	0.080
		Professional quality	0.667	Legal	0.014	4.75 ± 0.45	0.095
				Rules and regulations	0.023	4.83 ± 0.39	0.080
				Ethics	0.014	4.75 ± 0.45	0.095
				Professional attitudes	0.014	4.75 ± 0.45	0.095
				Responsibility	0.036	5.00 ± 0.00	< 0.001
				Code of conduct	0.030	4.92 ± 0.29	0.059
				Rights and Obligations of the Service Object	0.014	4.75 ± 0.62	0.130
				Humanistic care	0.023	4.83 ± 0.39	0.080
Knowledge	0.25	Activity of daily life	0.192	Knowledge of clean care	0.009	4.92 ± 0.29	0.059
				Knowledge of diet care	0.009	4.92 ± 0.29	0.059
				Knowledge of sputum care	0.009	4.92 ± 0.29	0.059
				Knowledge of sleep care	0.003	4.66 ± 0.65	0.139
				Knowledge of excretion care	0.004	4.75 ± 0.45	0.095
				Knowledge of mobile care	0.004	4.75 ± 0.62	0.13
				Care of incontinent-associated dermatitis	0.006	4.83 ± 0.39	0.08
				Knowledge of pressure injury	0.006	4.83 ± 0.39	0.08
		Prevention of nosocomial infections	0.192	Cleaning and disinfection of environment and articles	0.014	4.83 ± 0.39	0.08
				Garbage classification and management	0.034	4.92 ± 0.29	0.059
		Communication	0.134	Knowledge of communication	0.017	4.83 ± 0.39	0.080
				Knowledge of communicating with special service objects	0.008	4.75 ± 0.45	0.095
				Knowledge of dispute prevention	0.008	4.75 ± 0.45	0.095
		Safe and first-aid	0.104	Knowledge of occupational safety	0.008	4.75 ± 0.62	0.130
				Knowledge of protective appliances	0.011	4.83 ± 0.39	0.080
				Knowledge of fire safety	0.003	4.41 ± 0.79	0.179
				Knowledge of primary first aid	0.005	4.66 ± 0.65	0.139
		Vital sign	0.068	Knowledge of vital signs	0.017	4.66 ± 0.65	0.139
		Rehabilitation	0.068	General knowledge of rehabilitation	0.017	4.33 ± 0.77	0.179
		Hospice care	0.030	General knowledge of hospice care	0.005	4.25 ± 0.75	0.177
				Highlights of hospice care	0.012	4.42 ± 0.67	0.151
		Knowledge of medication	0.065	Knowledge of traditional Chinese medicines	0.003	4.33 ± 0.78	0.179
				Decoction methods and precautions of Chinese medicine	0.007	4.41 ± 0.79	0.179
				Knowledge of common medication care	0.007	4.41 ± 0.67	0.151
		Identification and care of symptoms	0.068	Knowledge of fever recognition and care	0.005	4.92 ± 0.29	0.059
				Knowledge of cough recognition and care	0.004	4.83 ± 0.39	0.080
				Knowledge of vomiting recognition and care	0.003	4.83 ± 0.39	0.080
				Knowledge of pain recognition and care	0.002	4.75 ± 0.45	0.095
				Knowledge of dizziness recognition and care	0.002	4.66 ± 0.65	0.139
				Knowledge of palpitations recognition and care	0.002	4.66 ± 0.65	0.139
		Common disease care	0.079	Knowledge of diabetes care	0.003	4.58 ± 0.67	0.145
				Knowledge of hypertension care	0.003	4.58 ± 0.67	0.145
				Knowledge of stroke care	0.006	4.66 ± 0.65	0.139
				Knowledge of COPD care	0.006	4.66 ± 0.65	0.139
				Knowledge of cancer care	0.002	4.50 ± 0.80	0.177
Skill	0.5	Diet care skill	0.050	Meal preparation skill	0.004	4.66 ± 0.65	0.139
				Assisted feeding	0.011	5.00 ± 0.00	< 0.001
				Post-meal observation and emergency aid	0.011	5.00 ± 0.00	< 0.001
		Clean care skill	0.095	Oral hygiene techniques	0.005	4.92 ± 0.29	0.059
				Shaving and nail trimming techniques	0.005	4.92 ± 0.29	0.059
				Facial, hand and foot cleansing techniques	0.005	4.92 ± 0.29	0.059
				Techniques for combing and shampooing hair in bed	0.005	4.92 ± 0.29	0.059
				Bedtime scrubbing technique	0.005	4.92 ± 0.29	0.059
				Assisted bathing technique	0.005	4.92 ± 0.29	0.059
				Perineal cleansing technique	0.005	4.92 ± 0.29	0.059
				Assistance with dressing technique	0.005	4.92 ± 0.29	0.059
				Patient’s unit cleaning techniques	0.002	4.83 ± 0.58	0.119
				Changing an occupied bed technique	0.009	5.00 ± 0.00	< 0.001
		Sleep care skill	0.095	Sleep promotion techniques	0.014	4.50 ± 0.67	0.150
				Assisting patients to fall asleep	0.033	4.50 ± 0.67	0.150
		Sputum care skill	0.095	Dorsal sputum evacuation technique	0.048	4.83 ± 0.39	0.080
		Excretion care skill	0.095	Assisting patients with toileting	0.008	5.00 ± 0.00	< 0.001
				Assisting patients with bedside continence technique	0.005	4.92 ± 0.29	0.059
				Enterostomy bag care technology	0.008	4.83 ± 0.58	0.119
				Diaper changing technology	0.013	4.92 ± 0.29	0.059
				Specimen collection technique (urine, feces)	0.013	5.00 ± 0.00	< 0.001
		Mobile care skill	0.095	Lying positioning	0.013	5.00 ± 0.00	< 0.001
				Body position change technique	0.015	5.00 ± 0.00	< 0.001
				Assisting patients to get in and out of bed	0.013	5.00 ± 0.00	< 0.001
				Patient transportation technology	0.006	4.92 ± 0.29	0.059
		Use and observation of auxiliary instruments	0.050	Use of mobility aids	0.005	4.83 ± 0.39	0.080
				Use of decompression devices	0.010	4.92 ± 0.29	0.059
				Use of ice and hot water bags	0.010	4.92 ± 0.29	0.059
		Disinfection and isolation	0.095	Immersion	0.010	4.58 ± 0.67	0.145
				Rubbing	0.019	4.66 ± 0.65	0.139
				Terminal disinfection	0.019	4.66 ± 0.65	0.139
		Communication skills	0.090	Communication skills	0.045	4.83 ± 0.39	0.080
		Patient safety skills	0.095	Prevention and emergency of falls/bed falls	0.010	5.00 ± 0.00	< 0.001
				Prevention and emergency treatment of	0.010	5.00 ± 0.00	< 0.001
				Prevention and emergency of aspiration	0.006	4.92 ± 0.29	0.059
				Prevention and emergency management of choking	0.006	4.92 ± 0.29	0.059
				Prevention and emergency treatment of pipe slip	0.006	4.92 ± 0.29	0.059
				Use of protective gears	0.004	4.75 ± 0.45	0.095
				Care for behavioral disorders	0.004	4.75 ± 0.45	0.095
		Self-protection skill	0.053	Hand hygiene	0.008	5.00 ± 0.00	< 0.001
				Putting on and taking off hat/gauze mask and gloves	0.008	5.00 ± 0.00	< 0.001
				Putting on and taking off protective clothing	0.004	4.92 ± 0.29	0.059
				Occupational exposure prevention and post practice	0.008	5.00 ± 0.00	< 0.001
		First aid skills	0.050	Emergency response to utility outages	0.003	4.50 ± 0.67	0.150
				Fire contingency skill	0.006	4.50 ± 0.67	0.150
				Basic life support	0.011	4.83 ± 0.39	0.080
				Heimlich maneuver	0.006	4.75 ± 0.62	0.130
		Rehabilitation exercise	0.039	Assisting body movement	0.004	4.66 ± 0.65	0.139
				Functional positioning	0.008	4.83 ± 0.39	0.080
				Assisting passive movement of limbs	0.008	4.83 ± 0.39	0.080

## Discussion

This study developed a competency-based pre-service training program for NAs, guided by the Competency Iceberg Model and structured across three core dimensions: knowledge, skills, and professionalism. The AHP was employed to assign weights to each domain. The findings showed that the skills domain carried the highest weight (0.50), particularly for practical competencies such as hygiene care, excretion care, sputum management, and mobility assistance. At the same time, professionalism accounted for one quarter of the framework, with communication and emotional management identified as important competency components. These findings show that competent NAs practice relies not only on the execution of routine care tasks but also on the professional attributes that underpin effective interpersonal interactions and emotional regulation. This pattern is consistent with the central proposition of the Competency Iceberg Model [28], which emphasises that sustained job performance depends on both observable competencies and deeper professional characteristics. By assigning relative weights to different competencies, the framework provides an evidence-informed basis for prioritising training content and allocating educational resources within resource-constrained pre-service training programmes.

Although the Kendall’s W values were relatively modest, this finding may reflect the evolving nature of the NAs role in China. Considerable variation remains in role positioning, workforce deployment, and training practices across regions and healthcare institutions [17], which may have contributed to differences in experts’ judgments regarding competency requirements and training priorities. Nevertheless, all indicators met the predefined inclusion criteria, the coefficients of variation remained below acceptable thresholds, and all AHP consistency tests were satisfied, suggesting that the level of consensus was sufficient for framework development.

The high weighting assigned to the skills dimension is consistent with the practice-oriented nature of the NAs role, in which direct care is central. This finding also reflects the broader international shift in nursing education towards competency-based and practice-oriented training [19]. In the Chinese healthcare context, the prioritization of practical skills may additionally reflect the current role positioning of nursing assistants, who are primarily responsible for direct daily living care rather than clinical decision-making or complex technical procedures. Consequently, at the third-level indicator level, practical competencies such as hygiene care, mobility assistance, sleep care, sputum clearance, and elimination care received relatively high weights. These activities represent the core, most frequently performed tasks in routine practice, and poor performance may increase the risk of adverse outcomes, including pressure injuries, infection-related complications, respiratory problems, falls, and musculoskeletal injuries among caregivers [35–37]. Experts therefore appeared to place greater emphasis on competencies that directly influence patient safety and quality of care during everyday caregiving. This finding suggests that practical competencies may warrant particular attention during programme implementation, with adequate opportunities provided for the development and assessment of core caregiving skills.

The knowledge and professionalism dimensions each received a weight of 0.25, indicating that both are important in supporting safe and high-quality care. Within the knowledge dimension, relatively high weights were assigned to activities of daily living care and hospital infection prevention and control. This is broadly consistent with international definitions of the core responsibilities of NAs [3, 11, 12, 38]. However, knowledge acquisition does not automatically translate into standardised practice. Effective knowledge transfer is closely associated with structured training of appropriate intensity [14].

This study further highlights the importance of professionalism within the competency framework for NAs. This finding is consistent with growing international attention to non-technical competencies in healthcare practice [39, 40]. Communication skills and emotion management received relatively high weights, indicating that experts increasingly recognise the importance of these competencies in NAs practice. NAs work on the frontline of patient care and frequently encounter patient suffering, family anxiety, disease deterioration, and end-of-life situations. These contexts require continuous emotional regulation and interpersonal interaction. Previous studies have shown that insufficient emotion management is associated with burnout, compassion fatigue, and reduced care quality [39–41]. Therefore, professionalism may be positioned as an essential component of NAs training rather than as an adjunct to technical skills training. Communication and emotional management competencies could be incorporated into scenario-based training and assessment activities to support the coordinated development of professional attributes and practical skills.

The training framework proposed in this study adopts a centralized and policy-driven approach. This model is similar to systems used in countries such as Singapore, where a unified national framework, such as the Skills Framework for Healthcare, helps ensure consistency in training and role definition [42]. In contrast, more decentralized models are used in countries such as the United States and Japan [13, 14, 43]. In the United States, federal regulations specify only minimum training requirements, while training program content is determined by individual states [14]. In Japan, training is mainly the responsibility of individual healthcare institutions, and no unified national standard has yet been established [43]. These institutional differences have shaped how the NAs role is positioned across countries. Singapore and Japan, although organised through different regulatory arrangements, both define NAs primarily as non-technical and supportive care workers [42, 43]. The framework developed in this study follows a similar pathway by emphasising standardised daily care tasks, which is consistent with current regulatory requirements in China. By contrast, in more decentralised systems such as the United States and Australia, NAs may undertake expanded roles and perform authorised technical tasks after advanced training [13]. These differences reflect variations in workforce strategies and regulatory traditions across countries. They should not be viewed as inherently superior or inferior; rather, each represents an adaptive choice within a specific national context. For China, a centralised framework may help address the current challenges, including fragmented training content and variable training quality, while still allowing local institutions to adapt implementation and evaluation methods according to available resources and regulatory requirements [13].

Future research should validate this framework in real-world healthcare settings through pilot and implementation studies. Evaluation should include both quantitative outcomes, such as competency assessment scores, patient safety indicators, and NAs job satisfaction, and qualitative evidence on implementation barriers and the experiences of trainers and trainees. Findings from such studies could inform further refinement of training delivery, including the use of blended learning, digital platforms, simulation-based training, and structured supervision, which may improve the effectiveness and accessibility of training [44]. The competency weights identified in this study may also guide the allocation of teaching time, particularly by prioritising core practical skills and supporting the development of a modular competency-based training system. Finally, comparative studies are needed to assess the effectiveness of this framework against existing training models and to provide evidence for its further refinement and wider implementation.

## Limitations

This study has several limitations. First, the use of an online modified Delphi method may have limited the depth of expert interaction and introduced potential information bias in qualitative feedback. Second, the development of the training program relied exclusively on expert opinion and did not incorporate the perspectives of key stakeholders such as NAs, patients, or family members. These groups may offer important insights into the relevance, feasibility, and acceptability of training content, as well as its impact on care quality and care experience. Third, although experts were recruited from multiple provinces, most were affiliated with tertiary hospitals in Eastern and Central China. This distribution may not fully reflect the training needs, resource constraints, and practice variations in primary care settings or less-developed regions, which may limit the generalizability of the findings.

## Conclusion

This study developed a standardized pre-service training program for NAs in China by integrating expert consensus through a modified Delphi method with quantitative prioritization using AHP. Grounded in the Competency Iceberg Model, the program provides a structured framework that integrates essential knowledge, practical skills, and key professional competencies. The AHP-derived weights offer an evidence-based rationale for prioritizing training content and allocating instructional resources. By aligning core training content with defined competency priorities, this framework may inform the development of more standardized national training approaches to NAs pre-service training while allowing adaptation to local contexts. Future research should focus on pilot implementation and empirical validation across diverse clinical settings.

## Data Availability

The datasets used and/or analyzed during the current study are available from the corresponding author on reasonable request.

## Abbreviations

NAs: Nursing assistants
AHP: Analytic hierarchy process
Ca: Coefficient of judgment
Cs: Degree of familiarity
Cr: Coefficient of authority
CV: Coefficient of variation
W: Kendall harmony coefficient
RR: Recovery rate
RI: Random consistency index
CI: Consistency index
SD: Standard deviation
